# Expression of Herpes Simplex Virus Thymidine Kinase/Ganciclovir by RNA *Trans*-Splicing Induces Selective Killing of HIV-Producing Cells

**DOI:** 10.1016/j.omtn.2017.03.004

**Published:** 2017-03-14

**Authors:** Carin K. Ingemarsdotter, Sushmita Poddar, Sarah Mercier, Volker Patzel, Andrew M.L. Lever

**Affiliations:** 1 Department of Medicine, Addenbrooke’s Hospital, University of Cambridge, Cambridge CB2 0QQ, UK; 2 Department of Microbiology & Immunology, Yong Loo Lin School of Medicine, National University of Singapore, 5 Science Drive 2, Singapore 117545, Singapore

**Keywords:** HIV, RNA splicing, RNA *trans*-splicing, gene therapy, exon replacement, herpes simplex virus thymidine kinase-ganciclovir, HSV-tk/GCV

## Abstract

Antiviral strategies targeting hijacked cellular processes are less easily evaded by the virus than viral targets. If selective for viral functions, they can have a high therapeutic index. We used RNA *trans*-splicing to deliver the herpes simplex virus thymidine kinase-ganciclovir (HSV-tk/GCV) cell suicide system into HIV-producing cells. Using an extensive in silico bioinformatics and RNA structural analysis approach, ten HIV RNA *trans*-splicing constructs were designed targeting eight different HIV splice donor or acceptor sites and were tested in cells expressing HIV. *Trans*-spliced mRNAs were identified in HIV-expressing cells using qRT-PCR with successful detection of fusion RNA transcripts between HIV RNA and the HSV-tk RNA transcripts from six of ten candidate RNA *trans*-splicing constructs. Conventional PCR and Sanger sequencing confirmed RNA *trans*-splicing junctions. Measuring cell viability in the presence or absence of GCV expression of HSV-tk by RNA *trans*-splicing led to selective killing of HIV-producing cells using either 3′ exon replacement or 5′ exon replacement in the presence of GCV. Five constructs targeting four HIV splice donor and acceptor sites, D4, A5, A7, and A8, involved in regulating the generation of multiple HIV RNA transcripts proved to be effective for *trans*-splicing mediated selective killing of HIV-infected cells, within which individual constructs targeting D4 and A8 were the most efficient.

## Introduction

More than 39 million people worldwide are infected with HIV, according to the World Health Organization. Although highly active antiretroviral therapy (HAART) is effective at suppressing viral replication, treatment cannot eliminate the virus because it resides latently in a population of immune cells referred to as memory T cells (reviewed by Van Lint et al.^1^). The existence of this reservoir, and the high mutation rate of HIV with the subsequent development of drug resistance,^2^ means that alternative treatments are needed that not only suppress viral replication but can also eliminate infected cells. Conventional therapies do not selectively target infected cells, and adverse drug effects are common. In addition, no currently licensed antiviral drugs are able to eliminate cells in which the virus is latent. Latency in HIV does not equate to permanent transcriptional silencing, and there is evidence that it is stochastic.^3^ Incomplete RNA transcripts may be found in cells that are not producing intact virus, and viral RNA-spliced products have been detected in resting CD4^+^ T cells isolated from HIV-infected patients on HAART.^4^ The viral RNA is thus an unexploited target to treat actively replicating virus and, at any one time, a significant proportion of latently infected cells. HIV uses alternative RNA splicing to process its genetic material into transcripts encoding different viral proteins (reviewed by Tazi et al.^5^). A recent study detected up to 109 different spliced HIV RNAs in HIV-infected T cells.^6^ The expression of these is tightly regulated, and when the HIV splicing process is modulated, viral replication and production is impaired.^7^, ^8^, ^9^, ^10^ Given its importance for the viral life cycle, HIV splicing is an attractive target for the development of novel antiviral therapies.^5^, ^11^, ^12^ Recent work shows that the small-molecule inhibitors digoxin^13^ and 8-azaguanine^14^ inhibit HIV replication by altering HIV alternative splicing,^13^, ^14^ and the compound ABX464 enhances HIV RNA splicing, thereby also compromising HIV replication.^15^

In addition to pharmacological inhibition of HIV, cell and gene therapies are attractive potential therapies against HIV that may lead to a more sustained control of viral rebound (reviewed by Hoxie et al.^16^ and Herrera-Carrillo et al.^17^). RNA *trans*-splicing is an elegant gene therapy method that has been used to correct acquired or inherited genetic disorders.^18^ It subverts the cellular RNA splicing machinery to exchange, through a *trans*-splicing reaction, a defective RNA transcript with a corrected mRNA molecule delivered in *trans.* In addition to correcting genetic mutations, RNA *trans*-splicing has been used to deliver cell death signals in order to specifically kill targeted cells (reviewed by Drude et al.^19^). RNA *trans*-splicing has been used to deliver the two-step *Herpes simplex* virus thymidine kinase/ganciclovir (HSV-tk/GCV) cell death system as a potential cancer therapy.^20^, ^21^, ^22^, ^23^, ^24^ HSV-tk acts by phosphorylating the pro-drug GCV, an analog of deoxyguanosine triphosphate, into an active compound, leading to chain termination during DNA replication and cell death (reviewed by Duarte et al.^25^). The HSV-tk/GCV system has been expressed in HIV-infected cells and proposed as a gene therapy against HIV.^26^, ^27^, ^28^, ^29^, ^30^, ^31^, ^32^ The extensive use of splicing by HIV to regulate its life cycle suggests that there are likely to be vulnerable splicing reactions susceptible to a *trans*-splicing approach. However, this has never previously been formally investigated using modern bioinformatic tools to identify those RNA species involved in individual splicing reactions that have structures favoring *trans*-splicing and that would be most effectively targeted.

We performed a comprehensive in silico analysis of HIV splicing and RNA structure to both find optimal targets and to design HIV binding domains in the *trans*-splicing constructs to, as near as possible, guarantee the specificity of the RNA *trans*-splicing reaction and facilitate the process. Using a combination of HIV splice site predictions, HIV RNA free energy calculations, and RNA secondary structure predictions together with an in-depth analysis of the literature, we were able to select a panel of ten 3′ and 5′ exon replacement constructs. These were then subject to testing for their ability to induce *trans*-splicing with transcripts from replicating virus. In those in which *trans*-splicing was confirmed at a molecular level, functional screening by MTT assay revealed four susceptible RNA target splice sites at which this approach led to specific killing of HIV-producing cells, with 3′ exon replacement being overall most efficient.

## Results

### In Silico Design of RNA *Trans*-Splicing Binding Domains and Selection of Target HIV Splice Sites

**Figure 1:**
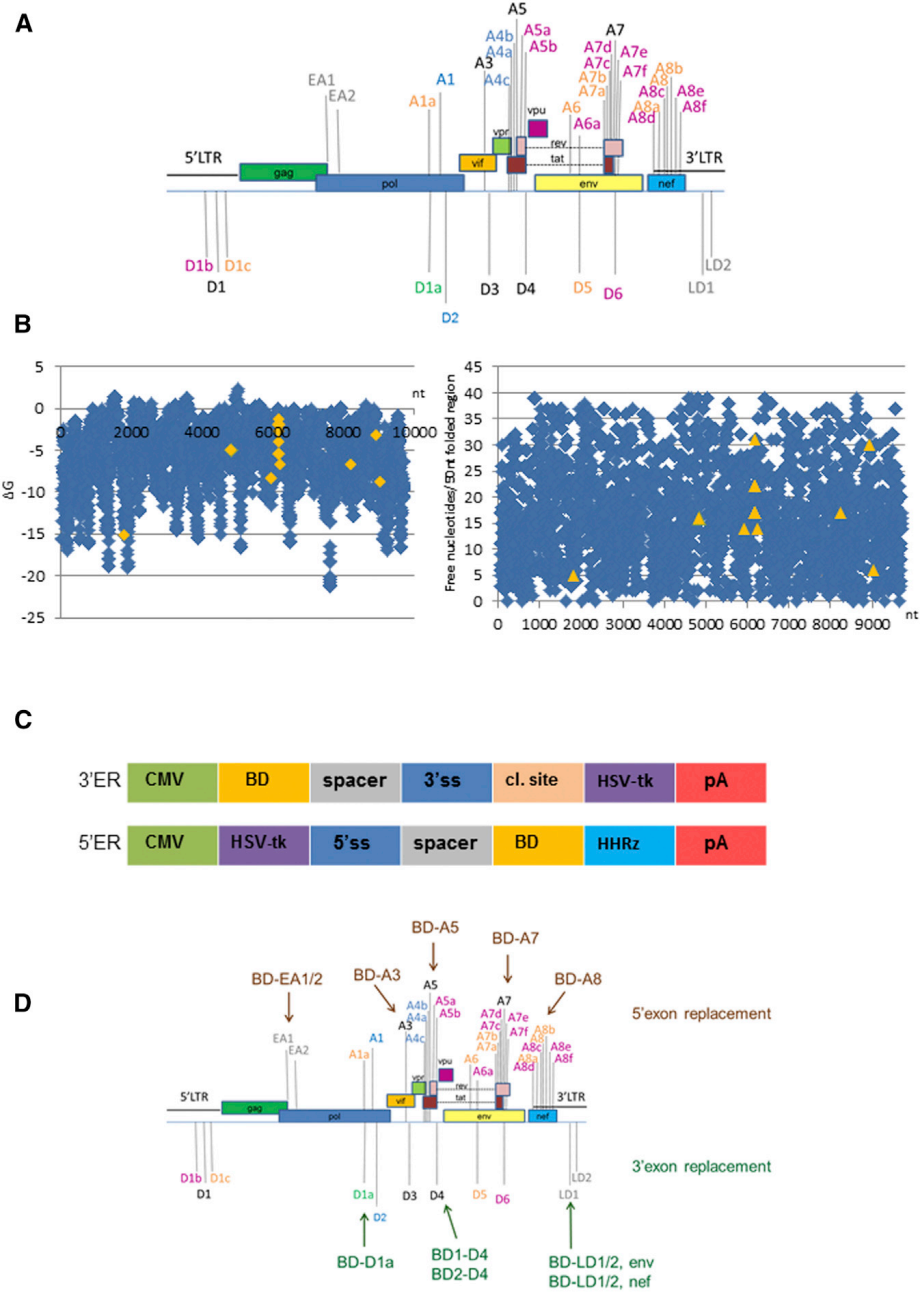
In Silico Analysis of HIV Splicing and Splice-Site Target Determination for RNA *Trans*-Splicing (A) Summary of HIV splice sites considered for targeting by RNA *trans*-splicing. Conserved splice donor/acceptor sites predicted by computer splice site predictions of pNL4.3 are shown in black.^87^ In green, previously reported cryptic splice donor site predicted by computer algorithms.^41^ In gray, late splice donor/early splice acceptor sites predicted in pNL4.3 by computer algorithms. In blue, conserved splice donor/acceptor sites not predicted in pNL4.3 by computer splice site predictions but previously published.^87^ In orange, previously reported cryptic splice sites not predicted in pNL4.3 by computer algorithms (A1a,^41^ A6,^50^, ^51^, ^52^ D5,^50^, ^51^ A7a, A7b,^88^ A8a, A8, A8b^89^). In purple, recently described splice donor and splice acceptor sites not predicted by computer algorithms,^6^ A5a.^6^, ^89^, ^90^ (B) Selection of binding domain regions using the software Foldanalyze (HUSAR). MFE analysis of pNL4.3 throughout the complete reverse complement pNL4.3 genome per 50 nt folded region (left). Number of free nucleotides per 50 nt folded region of the complete reverse complement pNL4.3 genome (right). Target regions of binding domains selected for further design are shown in orange. (C) Maps of CMV promoter-driven RNA *trans*-splicing cassettes. 3′ exon replacement (top) and 5′ exon replacement (bottom) cassettes are shown. BD, binding domain; spacer, spacer sequence; 3′ss, 3′ splice site domain; cl. site, P2A protein cleavage site^91^; HSV-tk, herpes simplex virus thymidine kinase gene; pA, polyA tail; HHRz, hammerhead ribozyme sequence. (D) Summary of selected HIV splice sites for targeting by 5′ exon replacement (top) or 3′ exon replacement (bottom). HIV splice site D4 was targeted with two different binding domain sequences (BD1-D4 and BD2-D4), and LD 1 and 2 (LD1 and LD2 respectively) were targeted with two BD-LD1/2 cassettes driven from either HIV Env or HIV Nef translational start sites.

HIV splice sites were predicted using computer software as described in Materials and Methods. The splice site prediction analysis of pNL4.3 gave similar results with both software packages used (data not shown). Predicted splice site sequences were compared with those previously described in the literature, and the most favorable were selected for targeting by *trans*-splicing (Figure 1A). Splice sites that had previously been reported in the literature matching our computer predictions, or that we identified as present within pNL4.3, were considered. In addition, two groups of two computer-predicted HIV splice sites each referred to as early acceptor (EA) and late donor (LD) sites were included, comprising the sites EA1 and EA2 (EA1/2) and LD1 and LD2 (LD1/2), respectively (Figure 1A).

**Figure 2:**
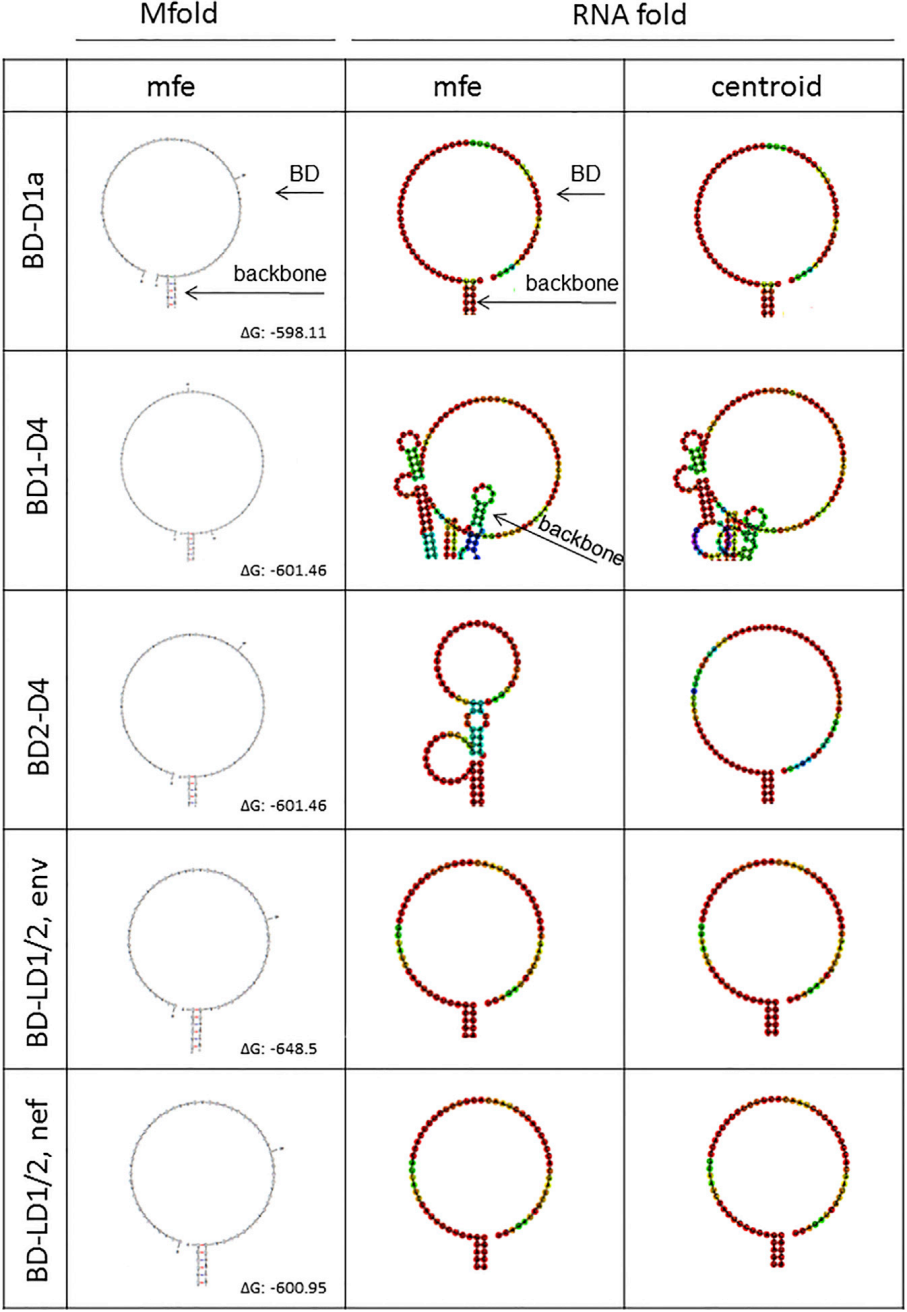
RNA Secondary Structure Predictions of Designed 3′ Exon Replacement HIV Binding Domains RNA secondary structures of 3′ exon replacement cassettes containing HIV binding domains were predicted using Mfold and RNAfold web servers. MFE RNA secondary structures of HIV binding domain regions predicted in Mfold are shown (left column). RNAfold secondary structure predictions showing MFE (middle column) and centroid folds are shown (right column). RNA secondary structures of HIV binding domains with only a small region of the backbone plasmid are shown for clarity. Predicted free energies for the complete fold (binding domain and backbone cassette) are shown as ΔG.

**Figure 3:**
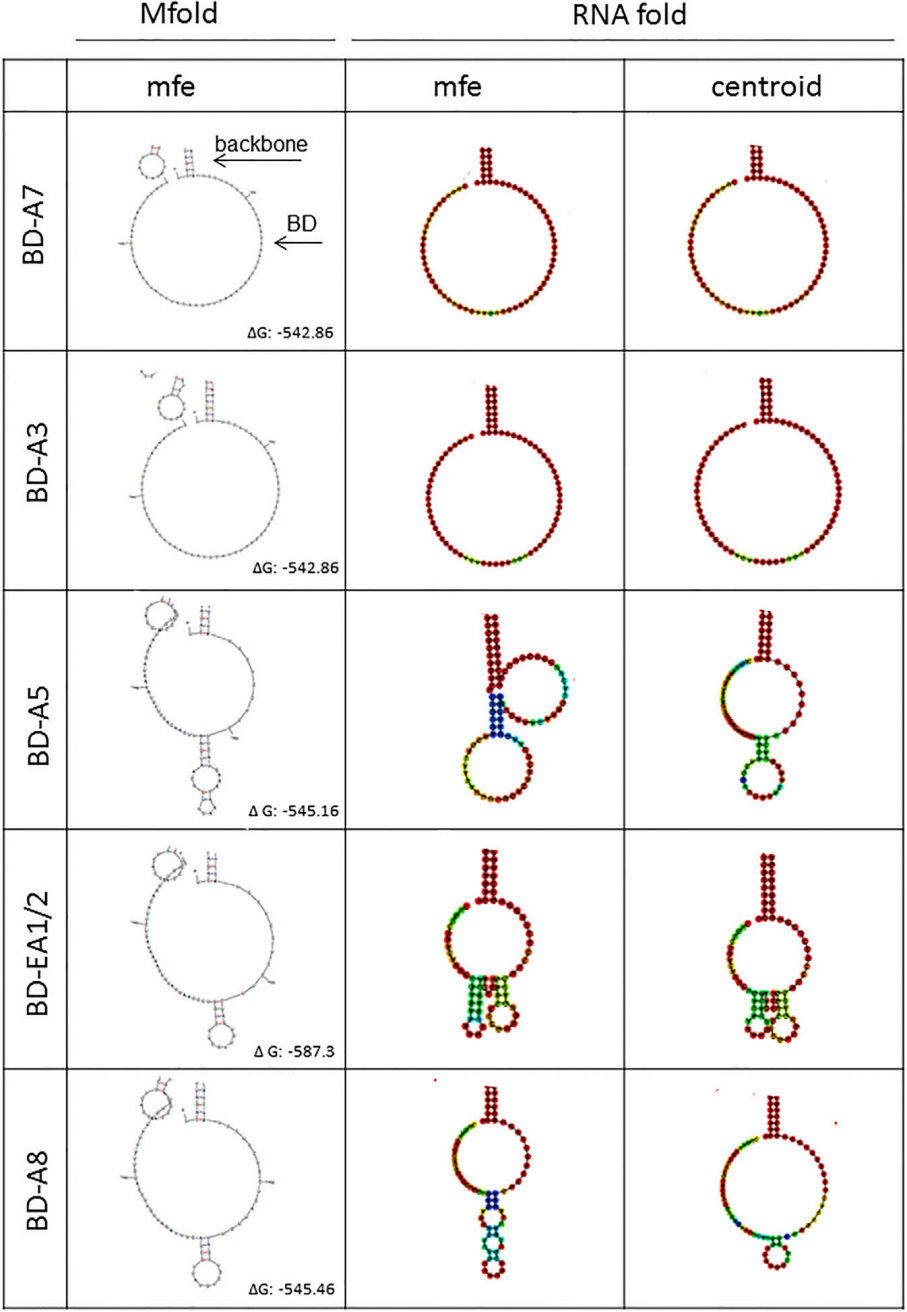
RNA Secondary Structure Predictions of Designed 5′ Exon Replacement HIV Binding Domains after HHRz RNA Self-Cleavage to Release the Binding Domain RNA secondary structures of 5′ exon replacement cassettes containing HIV binding domains were predicted using Mfold and RNAfold web servers. MFE RNA secondary structures of HIV binding domain regions predicted in Mfold are shown (left column). RNAfold secondary structure predictions showing MFE (middle column) and centroid folds (right column). RNA secondary structures of HIV binding domains with only a small region of the backbone plasmid are shown for clarity. See Figure S1 B for a schematic diagram of HHRz self-cleavage.

RNA folding energies of the complete reverse complement HIV pNL4.3 genome in 50 nucleotide windows from 5′ to 3′ were calculated (Figure 1B, left). The number of free nucleotides per folding window of selected binding domains subjected to further design is shown (Figure 1B, right). Selected structures were then refolded in the context of the backbone 3′ or 5′ exon replacement cassette sequences to confirm lack of interference by flanking regions (Figure 1C), and final structures were generated by further sequence modification. From this detailed in silico analysis, three HIV splice donor sites, D4, D1a, and LD1/2, were targeted with five 3′ exon replacement constructs, and five HIV splice acceptor site, EA1/2, A3, A5, A7, and A8, were targeted with 5′ exon replacement (Figure 1D). Final HIV binding domain structures folded in the backbone cassettes, all displaying predominantly unstructured binding domain regions, are shown for 3′ exon replacement in Figure 2 and for 5′ exon replacement in Figure 3. For clarity, only the binding domain structures are shown. Target regions are shown in Tables S1A and S1B, for 3′ exon replacement and 5′ exon replacement, respectively, and schematic diagrams of 3′ exon replacement and 5′ exon replacement are outlined in Figures S1A and S1B and of HSV-tk in Figures S2A and S2B.

### Sequence Conservation of HIV Binding Domain Target Regions

Given the high level of sequence variability of HIV, it was important to confirm that the sequences we used for the binding domains would target the maximum number of HIV strains. Sequence conservation of the HIV binding domain target regions was assessed using the AnaylzeAlign software.^33^ For the 3′ exon replacement construct BD2-D4, the maximum number of mutations of the HIV binding domain target region reached 32 in a maximum percentage of 21.6% from 3,944 aligned sequences, including all available HIV-1 sequences within the Los Alamos National Laboratory (LANL) filtered web alignment. With BD1-D4, this was reduced to 30 mutations in a maximum of 12.75% sequences from the consensus sequence. For the 5′ exon replacement constructs, the number of mutations was small, ranging from 7, 8, 6, to 33, in a maximum of 14.79%, 19.72%, 27.55%, and 9.92% for BD-A7, BD-A5, BD-A3, and BD-A8 in 4,632, 2,712, 2,445, and 4,553 sequences analyzed, respectively (Table S2). Of the binding domain target sequences, BD-A8 was the least conserved, with 33 mutations but with a maximum of 9.92% variants from 4,553 sequences analyzed. Interestingly, when sequence variation was instead analyzed compared with the consensus sequence for the major subtypes, a decrease in the number of mutations of the maximum percentage of sequences was seen in half of the constructs, which could be diminished in all of the constructs to very low levels when sequence variation within subtype group B was analyzed. The number of mutations ranged from 3 in BD-A7 to 9 in BD-A8, with the exception of BD2-D4 showing the least conserved binding domain target sequence, with 27 mutations in a maximum of 20.16% of the sequences within subtype group B. Remarkably, when comparing the HIV target sequences for BD1-D4, the number of mutations could be reduced from 30 in 12.75% when comparing all HIV-1 sequences in the alignment to 7 mutations in 20.44% within subtype B sequences. Similarly, the number of mutations was reduced for BD-A8 target region from 33 in 9.92% of sequences when comparing all HIV-1 sequences to 9 mutations in 14.55% of sequences within subtype B, suggesting that the majority of our HIV binding domains are located in regions with high conservation within HIV-1 subtype B viruses.

We next aligned our HIV binding domain target sequences in pNL4.3 with the consensus sequences for each binding domain target region within subtype B using BLAST.^34^ Needleman-Wunsch global alignments revealed sequence identities of 73% for BD1-D4, 55% for BD2-D4, 49% for BD-A3, 98% BD-A5, 96% for BD-A7, and 64% for BD-A8 compared with their respective subtype B consensus sequences, revealing that some of our binding domain target regions within pNL4.3 show some differences from the consensus sequence, although BD-A7 and BD-A5 show high levels of sequence identity.

### Screening and Detection of RNA *Trans*-Splicing Junctions in HIV-Producing Cells

**Figure 4:**
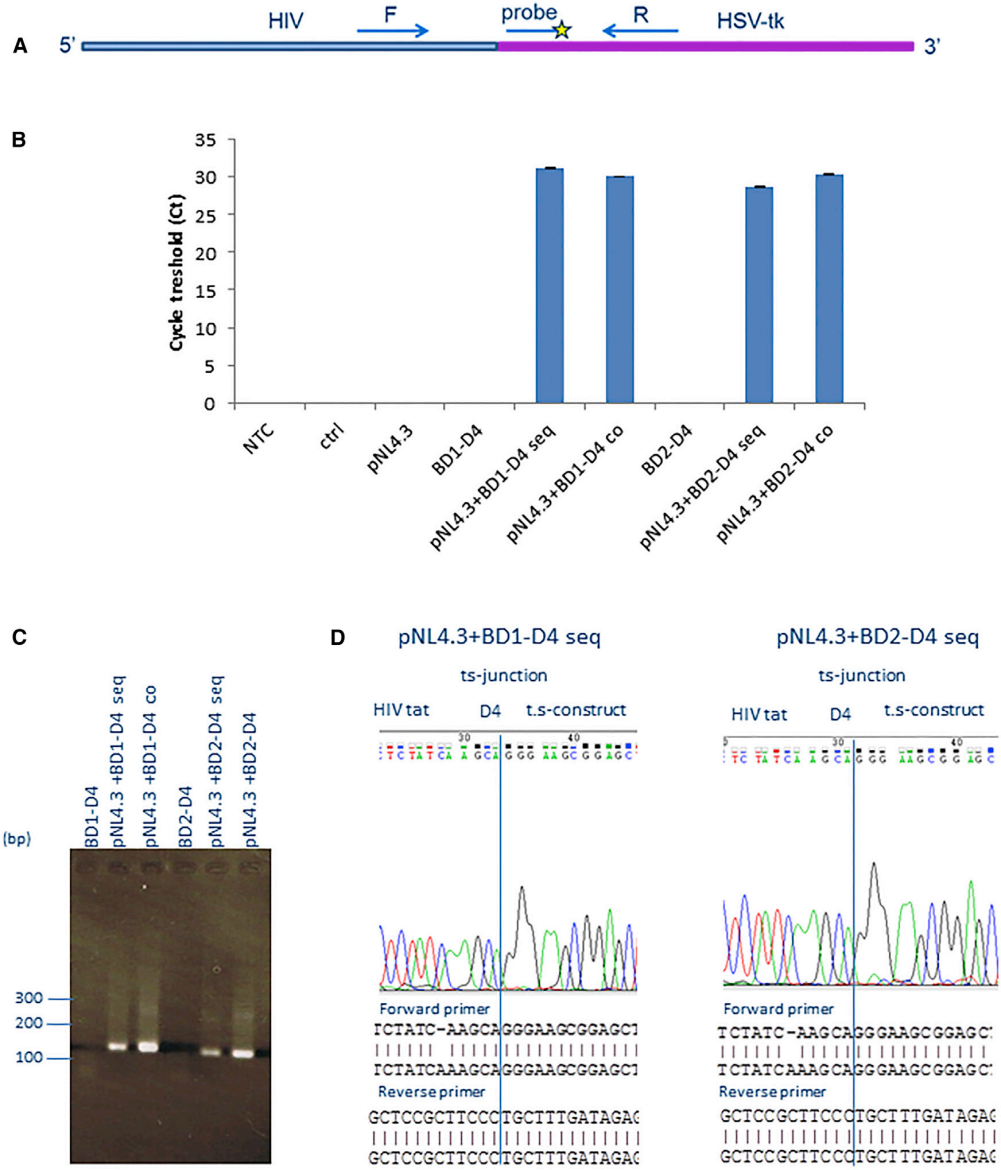
Confirmation of RNA *Trans*-Splicing between HIV and 3′ Exon Replacement Constructs (A) qRT-PCR primer and probe design to detect RNA *trans*-splicing amplicons. The forward primer is located in the HIV region and the reverse primer in the HSV-tk region. The PCR amplicon was detected with a probe in the HSV-tk region. (B) qRT-PCR data in cells transfected with the HIV proviral clone pNL4.3 followed by transfection with 3′ exon replacement construct BD1-D4 or BD2-D4. Cells were analyzed 48 hr post-transfection with the RNA *trans*-splicing constructs in sequentially transfected cells (seq) or co-transfected cells (co). (C) Confirmation of RNA *trans*-splicing by conventional PCR in sequentially (seq) or co-transfected cells (co). (D) Confirmation of RNA *trans*-splicing products by Sanger sequencing between HIV *tat* and HSV-tk constructs. BD1-D4 (left) and BD2-D4 (right). The error bars represent the standard deviation (STDEV) of average Ct values.

To test the activity of our 3′ exon replacement RNA *trans*-splicing constructs, we performed an initial screen in 293T cells. Cells were either co-transfected with the proviral clone pNL4.3 and the 3′ exon replacement constructs, or the latter was transfected 24 hr later. RNA *trans*-splicing should lead to the production of chimeric mRNAs; these were sought by qRT-PCR (Figure 4A). RNA *trans*-splicing amplicons were detected selectively in samples containing both HIV and RNA *trans*-splicing constructs BD1-D4 and BD2-D4, both in sequentially and co-transfected cells (Figure 4B). These could also be detected by conventional electrophoresis (Figure 4C); the PCR products were analyzed by Sanger sequencing, confirming junctional sequences (Figure 4D) between the HIV *tat* transcript and the *trans*-splicing construct.

**Figure 5:**
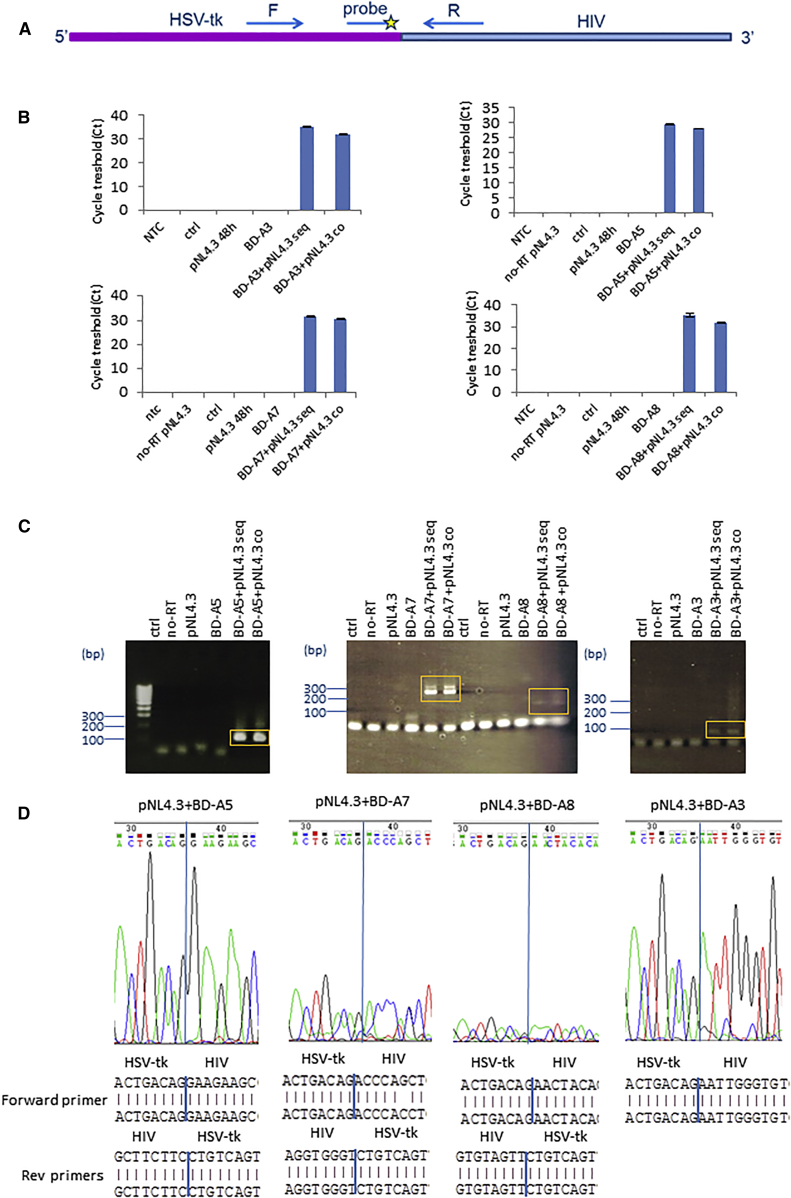
Confirmation of RNA *Trans*-Splicing between HSV-tk and 5′ Exon Replacement Constructs (A) qRT-PCR primer and probe design to detect RNA *trans*-splicing amplicons. The forward primer is located in the HSV-tk region and the reverse primer in the HIV region. The PCR amplicon was detected with a probe in the HIV region. (B) qRT-PCR data from cells transfected with the HIV proviral clone pNL4.3 followed by transfection with 5′ exon replacement constructs sequentially (seq) or after co-transfection of the two constructs (co). Top: data for BD-A3 and BD-A5 (left to right). Bottom: data for BD-A7 and BD-A8 (left to right). (C) Detection of HIV RNA *trans*-splicing products by conventional PCR. RNA *trans*-splicing was analyzed by with primers specific for BD-A5 (left), BD-A7 and BD-A8 (middle), and BD-A3 (right). PCR products highlighted in orange were PCR-purified and further analyzed by Sanger sequencing, as in (D). (D) Confirmation of RNA *trans*-splicing junctions by Sanger sequencing in sequentially transfected cells. Chromatographs showing sequence reads are included (top). PCR products as shown in (C) were analyzed by sequencing with a 5′ER forward primer and HIV-specific reverse primers. Sequencing data are shown from left to right for BD-A5, BD-A7, BD-A8, and BD-A3. RNA *trans*-splicing junctions are shown by blue lines on sequence chromatographs and sequence reads.

We next sought chimeric mRNA upon 5′ exon replacement. An analogous strategy to 3′ exon replacement was used to detect RNA *trans*-splicing amplicons in the presence of pNL4.3 and 5′ exon replacement constructs in either sequentially or co-transfected cells (Figure 5A). In four of five 5′ exon replacement constructs, BD-A3, BD-A5, BD-A7, and BD-A8, chimeric RNA transcripts could be detected between HSV-tk and HIV by qRT-PCR (Figure 5B). RNA *trans*-splicing amplicons could also be detected by conventional PCR and electrophoresis (Figure 5C), and Sanger sequencing confirmed junctional sequences (Figure 5D). Taken together, this demonstrates that 3′ or 5′ exon replacement between HIV and HSV-tk is feasible and that both generate the expected RNA *trans*-splicing products.

### Expression of HSV-tk by RNA *Trans*-Splicing Leads to Selective Killing in HIV-Producing Cells

**Figure 6:**
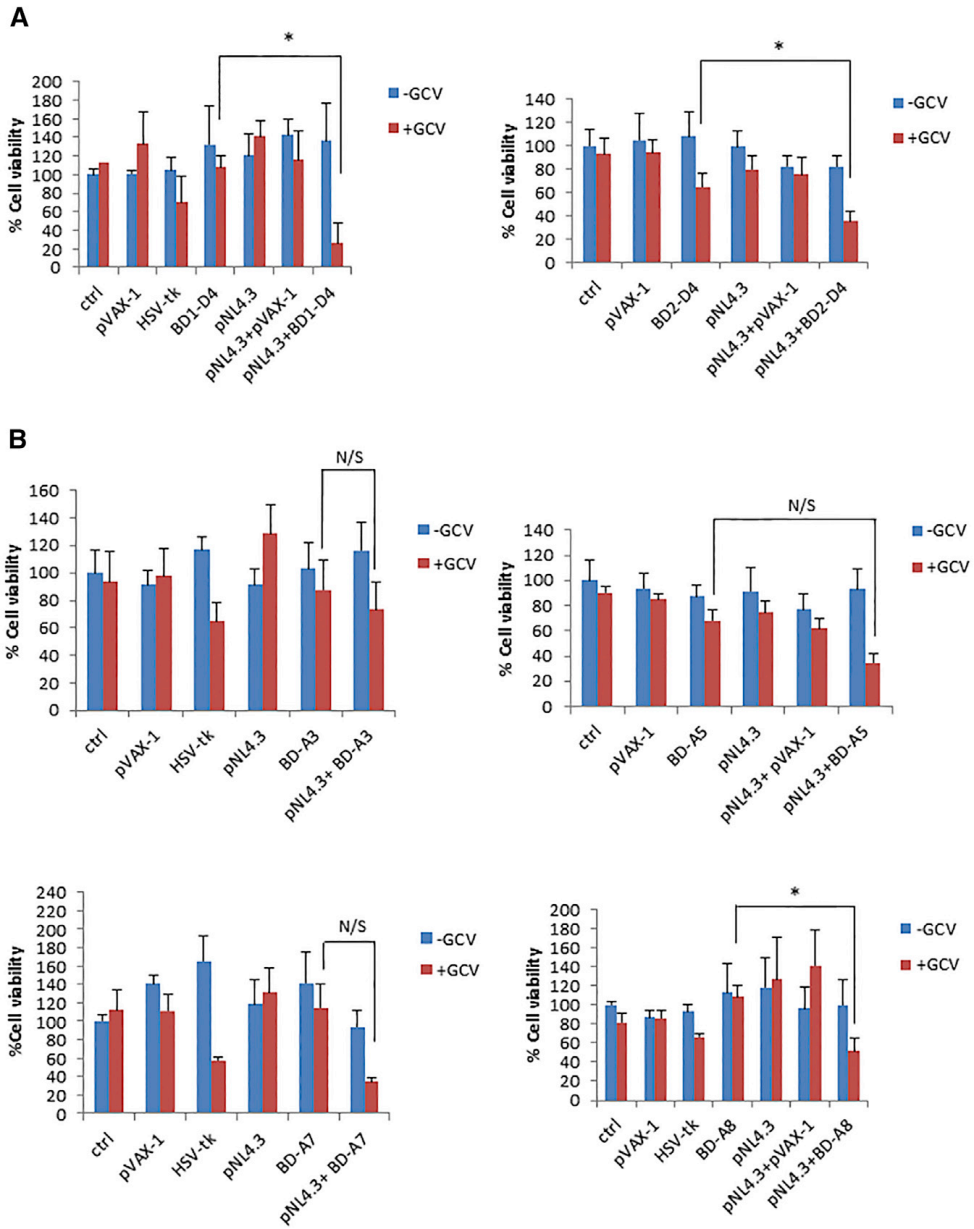
Cell Viability Screen of Exon Replacement Constructs in HIV-Producing Cells 293T cells were transfected with pNL4.3 and sequentially transfected with RNA *trans*-splicing constructs the following day. Cells were treated with one or two doses of GCV at a concentration of 100 μM, and cell viability was measured by the MTT assay 6 days after the initial transfection. Ctrl refers to untransfected 293T cells, pVAX-1 (empty vector backbone) was used as a negative control, and HSV-tk (full length HSV-tk) was included as a positive control. (A) 3′ exon replacement in HIV-producing 293T cells in the presence or absence of GCV. (B) Cell viability screen of 5′ exon replacement constructs in HIV-producing 293T cells in the presence or absence of GCV. BD-A3 (top left), BD-A5 (top right), BD-A7 (bottom left), and BD-A8 (bottom right).The data in (A) and (B) represent the average mean of representative experiments performed in multiple wells (three to eight wells). The error bars represents the SD of the average percentage cell viability. p values (two-tailed) are shown for “t test: two sample assuming unequal variances”; *p ≤ 0.05), where statistical significance was observed in each of three independent experiments.

The expression of HSV-tk protein dependent on the presence of the target HIV RNA sequence should lead to selective killing in the presence of ganciclovir (GCV). 293T cells were transfected with pNL4.3 and RNA *trans*-splicing constructs sequentially. Twenty-four hours later, cells and appropriate controls as described in the legend of Figure 6 were treated with one or two doses of 100 μM GCV on 2 consecutive days 24 hr apart, and cell viability was measured by the MTT assay 3 days later (6 days post-transfection with pNL4.3). 3′ exon replacement, *trans*-spliced HSV-tk, reduced cell viability in the presence of GCV. Compared with untreated controls (100% viability), the mean values of cell viability from representative experiments performed in multiple wells decreased to 26.2% for pNL4.3+BD1-D4 and 35.3% for pNL4.3+BD2-D4 (Figure 6A). Cell viability of cells transfected with pNL4.3+BD1-D4 compared with BD1-D4-transfected cells in the presence of GCV was also statistically significantly different (p ≤ 0.05) in each of three independent experiments, with a reduction in cell viability in the presence of pNL4.3. Similarly, this was also observed with pNL4.3+BD2-D4 compared with BD2-D4-transfected cells in the presence of GCV (p ≤ 0.05 in each of three independent experiments). A much smaller although still significant reduction in cell viability was seen in cells transfected with BD2-D4 alone compared with untransfected cells treated with GCV, from 92.8% to 64.9% (p = 0.0035).

The effect of HSV-tk/GCV on cell viability was also investigated using 5′ exon replacement. 5′ exon replacement reduced cell viability in the presence of GCV to 34.61% for pNL4.3+ BD-A7, 34.93% for pNL4.3+BD-A5, 51.63% for pNL4.3+BD-A8, and 73.51% for pNL4.3+BD-A3, compared with control cells (Figure 6B). When cell viability was assessed as above in cells transfected with pNL4.3+RNA *trans*-splicing construct, compared with single-transfected cells solely expressing the RNA *trans*-splicing construct, both in the presence of GCV, a reduction in cell viability for pNL4.3+BD-A3, pNL4.3+BD-A7, pNL4.3+BD-A5, and pNL4.3+BD-A8 was observed, of which pNL4.3+BD-A8 reached statistical significance in each of three independent experiments (p ≤ 0.05). BD-A7 and BD-A5 were effective in reducing cell viability in the presence of pNL4.3, and BD-A3 had a modest effect, although for these constructs this did not reach statistical significance in all three independent experiments. The 3′exon replacement construct BD1-D4 was the most efficient of our panel of RNA *trans*-splicing constructs. To confirm that RNA *trans*-splicing activity is not restricted to the HIV-1 proviral clone pNL4.3, we tested BD1-D4 against the HIV-1 proviral clone HXB2 (SVC21). A significant reduction in cell viability was observed in the presence of the RNA *trans*-splicing construct BD1-D4 in HXB2-producing cells compared with cells transfected with BD1-D4 alone in the presence of GCV (p < 0.001), demonstrating that induction of cell death by RNA *trans*-splicing is not restricted to the proviral clone pNL4.3 (Figure S3).

## Discussion

HIV RNA splicing is central to the HIV viral life cycle. Three classes of mRNA transcripts are generated in infected cells: unspliced, partially spliced, and multiply spliced mRNAs. HIV uses alternative splice donor and acceptor sites in different combinations to generate a large repertoire of mRNA transcripts in a highly regulated fashion. The multiply spliced transcripts are produced early during infection to generate the *tat*, *rev*, and *nef* transcripts encoding the regulatory proteins Tat, Rev, and Nef. Tat drives transcription by binding to TAR in the 5′LTR, and Rev mediates nuclear export of the partially spliced and unspliced transcripts by binding to the Rev responsive element (RRE) within the *tat*/*rev* intron, leading to further production of viral proteins in the cytoplasm (reviewed by Tazi et al.^5^).

The central importance of splicing to HIV has led to exploration of possible ways to interfere with the process as a way to inhibit viral replication and control viral growth (reviewed by Tazi et al.^5^). Previous approaches have been largely empirical and have not used a detailed and extensive bioinformatic analysis to optimize the targeting. We have now refined this approach taking advantage of sophisticated predictive and RNA structural software and performing an extensive in silico analysis of HIV splice sites with the aim of HIV dependent expression of the HSV-tk/GCV cell suicide system by RNA *trans*-splicing. The specificity of the RNA *trans*-splicing reaction is guaranteed by having complementary sequences binding to the target pre-mRNA. Binding domains are generally based on target sequence (reviewed by Walsh^35^), but optimization of binding domains to include stem-loop structures has been tested,^36^ and fluorescence-based screens of binding domain libraries have been developed.^37^, ^38^, ^39^ In addition to the complementary nucleotide sequence, we considered RNA structure as an important element for efficient RNA *trans*-splicing. It was previously demonstrated by Patzel and Sczakiel^40^ that the secondary structure of artificial antisense RNA is important for efficient annealing to target RNA and that target binding represents the rate-limiting step of antisense RNA-triggered inhibition of gene expression. A fast annealing rate to the target region was shown to correlate with terminal unpaired nucleotides.^40^ Taking all of these factors into consideration, HIV binding domains were designed on the basis of stepwise minimum free energy (MFE) calculations. Using our approach, we confirmed successful RNA *trans*-splicing in six of ten designed constructs demonstrating use of the HIV donor site D4 and acceptor sites A3, A5, A7, and A8, all of which were confirmed by PCR and sequencing.

A comprehensive analysis of HIV splicing patterns by Ocwieja et al.^6^ revealed more than 100 spliced mRNA species. Of the donor splice sites identified (Figure 1A; Table S1), D4 that we targeted is one of the major conserved HIV splice sites used in vivo to generate the *vpr*, *tat*, *rev*, *nef*, and two novel transcripts. It is most commonly used in combination with the A7 splice acceptor site, followed by A8c, a recently identified site.^6^

We also confirmed RNA *trans*-splicing at HIV splice acceptor sites. The HIV splice acceptor site A3 is used to generate the *tat* transcripts and is most common used in combination with the D1 major splice donor, followed by D2 and then D3.^6^ Similarly, splicing at the HIV acceptor site A5 that generates the *env*/*vpu*, *rev*, and *nef* transcripts is most frequently used in combination with splice donor site D1, followed by D3 and D2. HIV splice acceptor site A7 generates the *vpr*, *tat*, *rev*, *nef*, and recently identified novel transcripts.^6^ In addition, A7 mainly splices onto donor site D4 followed by D1. HIV acceptor site A8 is a cryptic splice site splicing mainly onto D4, but low levels of splicing onto D1 and D1b has been shown.^6^ Although we were able to confirm RNA *trans*-splicing to the splice acceptor sites A5, A7, A3, and A8, we cannot exclude that low levels of RNA *trans*-splicing events are occurring at proximal acceptor sites. An additional band was observed when RNA *trans*-splicing amplicons were analyzed by conventional PCR with BD-A7 in the presence of HIV (Figure 5C). The A7 cluster of splice sites, A7c, A7d, A7, A7e, and A7f, are located within close proximity and with the reverse primer used in the PCR, whose target sequence is located downstream of A7f, it would be possible to amplify RNA *trans*-splicing products arising from splicing events occurring at proximal splice sites upstream of A7, which would give rise to a PCR amplicon of increased size. This could be observed, but the main component in the sequencing analysis revealed splicing from A7. Similarly, with BD-A5 and BD-A8, we cannot exclude low levels of RNA *trans*-splicing in the presence of HIV from the proximal A5a and A5b site in addition to A5 and from the A8 cluster of splice sites A8a, A8b, A8e, and A8f, respectively. Again, the main components in the sequencing analysis revealed splicing from A5 and A8. Taken together, this suggests that RNA *trans*-splicing events at the A5, A7, and A8 clusters of splice sites are dominant despite proximal splice sites being present.

We were not able to confirm RNA *trans*-splicing to the D1a splice donor site, which is an infrequently used cryptic site, but is suggested to have a role in RNA stability.^41^ Nor could we confirm use of EA sites designated EA1/2 or LD sites LD1/2 identified by our in silico predictions. However, these are putative sites that may not be favorable or may perhaps be silenced by splicing silencing regulatory domains. It is intriguing that these sites could not be activated even when RNA *trans*-splicing constructs provide splicing domains delivered in *trans*, possibly suggesting that they are tightly suppressed. No HIV splicing silencing domains have so far been identified in the vicinity of these predicted EAs and LD sites, suggesting that there may be additional HIV splice site silencing regulatory domains in the HIV genome.

Of the six constructs confirmed to *trans*-splice onto HIV, five led to cell killing in 293T cells in the presence of HIV. The 3′ exon replacement RNA *trans*-splicing candidates (BD1-D4 and BD2-D4) targeting HIV splice sites D4 are good candidates for further study. Of the 5′ exon replacement constructs, BD-A7, BD-A5, and BD-A8 reduced cell viability, of which BD-A8 was the most effective. Splicing to the D4 site by 3′ exon replacement with BD1-D4 gave the most pronounced reduction in cell viability. HIV splice donor site D4 has a high usage, second only to the major HIV splice donor site D1, onto which all RNA transcripts splice.^6^ Recently, Sherrill-Mix et al.^42^ observed chimeric RNA transcripts between HIV RNA and host-cell RNA in infected cells spliced from the D4 site onto cellular acceptor sites, and RNA *trans*-splicing from D4 to upstream acceptors has recently been observed, suggesting *trans*-splicing between different HIV RNA transcripts,^43^ which is in agreement with our finding that splicing in *trans* from D4 is feasible. In cells transfected with BD2-D4 alone, we observed a small reduction in cell viability, possibly due to non-specific binding of the binding domain to cellular pre-mRNA targets. The 3′ exon replacement constructs BD1-D4 and BD2-D4 have overlapping binding domains, but the binding domain nucleotide sequence of BD1-D4 binds 53 nucleotides upstream of BD2-D4 and is 17 nt longer, suggesting that increasing the length of the binding domain may enhance the specificity of the RNA *trans*-splicing reaction, which is in agreement with others.^44^ Indeed, of the two binding domains targeting D4, BD1-D4 was the most efficient construct in reducing cell viability specifically and, notably, the only binding domain with a length greater than 50 nucleotides. Importantly, the efficiencies of these two binding domains in reducing viability are directly comparable, as the expression of HSV-tk is driven from the same Tat translational start codon. The association of the longest binding domain with the greatest effect on GCV-mediated, HIV-dependent cell killing may be significant.

Both donor site D4 and acceptor site A3 are involved in generating the different *tat* RNA transcripts. D4 is used to generate the completely spliced *tat* RNA transcripts and A3 for both the completely spliced and partially spliced *tat* transcripts.^6^ Within the pNL4.3 genome, these sites are located 267 nt apart, and there are multiple splice site regulatory domains located between them.^45^ Although both sites are involved in generating Tat transcripts and are close together, we were not able to detect a significant reduction in cell viability with BD-A3 targeting the A3 site. During the preparation of this manuscript, Emery et al.^43^ reported that splice acceptor A3 is used to low frequency in pNL4.3. In addition, the HIV binding domains BD-A3 and BD2-D4 bind 946 nt upstream of splice site A3 and 149 nt downstream of D4 respectively, so it is possible that the distance between the binding domain target sequence and the splice site targeted is important. This may be an important consideration for *trans*-splicing binding domain design in general, as previously suggested.^46^ Intriguingly, BD-A3 targeting splice acceptor site A3 and BD-D1a targeting the cryptic splice donor site D1a have overlapping binding domain sequences, but RNA *trans*-splicing junctions could not be detected from D1a, confirming that the binding domain does not confer RNA *trans*-splicing per se.

In vivo, the conserved HIV splice acceptor sites included in our screen are used with the following frequency: A7 > A5 > A3 > A8 in the HIV-1 isolate 89.6.^6^ No clear correlation was seen between splice site usage and reduction in cell viability, although they are all conserved and widely used sites, except A8, a cryptic site.^6^

On the basis of previous findings from antisense RNA design,^40^ we selected and designed HIV binding domain sequences to have a high degree of free nucleotides and unstructured regions by in silico RNA secondary structure predictions, aiming for fast annealing rates. We also compared our binding domain candidates with previously published SHAPE reactivity data of the complete HIV genome,^47^ which revealed that our binding domains are targeted to regions containing highly reactive and thus predicted unpaired nucleotides, in agreement with our in silico predictions. The 5′ exon replacement binding domains BD-A5, BD-EA1/2, and BD-A8 contain small structural domains with hairpin loops, although the majority of nucleotides are free and unstructured. These hairpins were difficult to resolve; nevertheless, BD-A5 and BD-A8 had *trans*-splicing activity, suggesting that they had little effect on *trans*-splicing efficiency.

We investigated HIV binding domain target sequence variation and compared the number of mutations in the maximum percentage of sequences between sequences from all HIV-1 groups, the major subtypes, or within subtype B. The majority of the HIV binding domain targets are located in regions with a low maximum percentage of sequence variants, ranging from 9.92%–27.55% when all groups were included in the analysis. Sequence variation could be reduced when comparing HIV binding domain target sequences in the major subgroups and even further within subtype B. Interestingly, a recent study highlighted the nucleotide genomic diversity across the HIV genome at the large-scale population level,^48^ and Li et al.^48^ demonstrated that the nucleotide genomic diversity between HIV-1 groups was 37.5%, 14.7% between HIV-1 subtypes, and 8.2% within the different HIV-1 subtypes, which supports our findings that fewer variants were present in the maximum percentage of sequences within subtype B. Global sequence alignments of our binding domain target sequences in pNL4.3 with their respective subtype B consensus sequences revealed that in some cases, overall sequence identity was moderate between pNL4.3 and the subtype B consensus sequence, ranging from 49%–73%. Although it would be ambitious to claim that a single binding domain sequence would be universally applicable for *trans*-splicing, our data suggest that targeting individual subtypes such as subtype B with a single construct is highly feasible on the basis of consensus sequences. Developing HIV-1 therapeutics on the basis of consensus sequences has previously been suggested for HIV-1 vaccine development^49^ and would be an attractive and pragmatic approach for the development of next-generation HIV-1 RNA *trans*-splicing binding domains.

Despite the similarity in BD1-D4 HIV-1 binding domain target sequences between pNL4.3 and HXB2, these are distinct lab strains and show differences in splice site usage, HXB2 for example having an additional splice acceptor site A6,^50^, ^51^, ^52^ and the splice donor site D5,^50^, ^51^ which was not predicted in our in silico analysis in pNL4.3. Notwithstanding these differences, we were able to confirm a significant reduction in cell viability upon RNA *trans*-splicing with BD1-D4 in the HXB2 clone SVC21, indicating that it is feasible to target HIV-1 strains with some variations in splice site usage and that this did not affect RNA *trans*- splicing at the conserved HIV splice donor site D4 site.

The expression of HSV-tk lead to cell death in HIV-producing cells in the presence of GCV. Our 3′ exon replacement constructs lack the first translational initiation codon of HSV-tk and are designed so that an upstream in-frame HIV-1 translational initiation codon initiates HSV-tk expression. Intriguingly, HSV-tk has an unusual mechanism for translational initiation with additional translational initiation sites located downstream of the first ATG start codon, generating additional HSV-tk polypeptides,^53^, ^54^, ^55^ with an observed increase in usage when the first ATG is mutated.^56^, ^57^ We cannot exclude that some of the HSV-tk-induced cell death observed in the absence of HIV-1 stems from HSV-tk peptide expression driven from downstream translational initiation codons or other off-target effects (reviewed by Berger et al.^58^). Splicing in *cis* and protein expression from putative translational initiation codons within RNA *trans*-splicing molecules has previously been reported.^59^ Future work will aim at investigating the relative contribution of different HSV-tk peptides in HSV-tk/GCV-mediated cell death and potential off-target effects.

HSV-tk/GCV has been widely used in gene therapy to induce cell killing of malignant cells in a number of different cancers, in vitro and in vivo, with promising pre-clinical data (reviewed by Karjoo et al.^60^). HSV-tk-activated GCV has been shown to induce cell death through cellular DNA damage and apoptosis,^61^, ^62^ but non-apoptotic death has also been observed.^63^, ^64^, ^65^, ^66^ HIV infection leads to T cell depletion through apoptosis in permissive cells (reviewed by Février et al.^67^). However, the majority of HIV-driven CD4^+^ T cell depletion in lymphoid tissue occurs through pyroptosis of nonpermissive cells,^68^ with cell-to-cell viral spread being a crucial factor.^69^ By eliminating the population of HIV-producing cells through HSV-tk/GCV-induced cell death, it is reasonable to hypothesize that T cell depletion by pyroptosis may be reduced. Further research is needed to explore this, and to elucidate the precise mechanism of HSV-tk/GCV-induced cell death in the context of HIV infection.

HSV-tk/GCV-mediated gene therapy has reached phase I/II clinical trials and been shown to be safe, although with limited clinical efficacy. The vector system most widely used for HSV-tk/GCV cancer gene therapy is based on adenoviral vectors (reviewed by Karjoo et al.^60^), but because of the lack of Coxsackie and adenovirus receptor (CAR) on leukocytes,^70^, ^71^, ^72^ and the recent observation that Ad5-specific T cells are more susceptible to HIV,^73^ a conventional adenoviral-based vector may not easily be translatable to T cell-targeted HIV RNA gene therapy. Lentiviral vectors may be better candidates. Lentiviral-mediated gene transfer of RNA therapeutics against HIV has been studied both in pre-clinical studies in vivo and in clinical trials (reviewed by Hoxie et al.^16^). In addition, lentiviral-mediated gene delivery of the HSV-tk/GCV suicide system has been used in HIV-infected cells^32^ and to control graft-versus-host disease in a phase I/II clinical trial in patients with leukemia.^74^ Recently, it has been shown that HIV can infect cells of the hematopoietic lineage in vivo,^75^ and novel lentiviral vectors targeting CD34^+^ hematopoietic progenitor cells have been described.^76^ Bone marrow gene therapy is an attractive technology for HIV antiviral therapies (reviewed by Herrera-Carrillo et al.^17^), and lentiviral-mediated gene transfer of bone-marrow cells has been demonstrated to be feasible in patients,^77^, ^78^, ^79^ which could be a potential delivery method for RNA *trans*-splicing technology in HIV-infected individuals. Recently, Zhou et al.^80^ described the generation of an engineered lentiviral vector that can selectively transduce memory CD4^+^ T cells. Thus, in the future, it may be feasible to deliver RNA *trans*-splicing gene therapy systemically. Encouragingly, GlaxoSmithKline (GSK) recently received positive recommendation for market authorization of a gene therapy from the Committee for Medicinal Products for Human Use (CHMP), followed by marketing authorization for use within the European Union by the European Commission. The gene therapy constitutes autologous CD34^+^ cells transduced with a retroviral vector to express adenosine deaminase (ADA), and is targeted to treat severe combined immunodeficiency due to adenosine deaminase deficiency (ADA-SCID).^81^ Our future work will focus on targeting latent transcripts which may contain the major splice donor site D1 and generating RNA *trans*-splicing cassettes in suitable vectors for delivery into CD4^+^ T cells for use in in vitro and in in vivo studies.

To conclude, expressing HSV-tk/GCV by RNA *trans*-splicing is an efficient means to selectively induce cell death in HIV-producing cells. RNA *trans*-splicing by either 3′ exon replacement targeting splice donor site D4 or 5′ exon replacement targeting splice acceptor site A8 were the most efficient HIV splice sites to target. A detailed bioinformatic approach can facilitate design of effective *trans*-splicing constructs.

## Materials and Methods

To develop RNA *trans*-splicing vectors targeting HIV RNA splice sites, we used the HIV proviral clone pNL4.3 as a template sequence. HIV splice sites of this clone nt 1–9,709 (GenBank: AF324493.2) were predicted using the Splice Site Prediction by Neural Network Server within the Berkeley *Drosophila* Genome Project (http://www.fruitfly.org/seq_tools/splice.html),^82^ with minimum scores for 5′ and 3′ splice sites at 0.4. The probability for cryptic splice site activation was predicted for pNL4-3 with the CrypSkip software within the Bioinformatics HUSAR server, German Cancer Research Centre (https://genome.inet.dkfz-heidelberg.de/husar/hs_home.html). HIV binding domains were designed on the basis of stepwise MFE calculations of the pNL4-3 genome. The RNA folding energies for the reverse complement of pNL4-3 nt 1–9,709 was predicted with the Foldanalyze software within the Bioinformatics HUSAR server, with a window size of 50 and a step size of 1. Potential regions of HIV binding domains with high free energy and a large number of unpaired bases in the vicinity of predicted and selected HIV splice sites were subjected to MFE RNA secondary structure predictions using Mfold (http://mfold.rna.albany.edu/?q=mfold/RNA-Folding-Form),^83^ with an upper boundary on the number of computing foldings set to 1, and the percentage suboptimality number to 5. The MFE fold and partition function were predicted and calculated with the RNAfold web server (http://rna.tbi.univie.ac.at/cgi-bin/RNAfold.cgi). Structures displaying both a large number of unpaired nucleotides and similar predicted structures with both software packages were selected for further design. Selected structures were then refolded in the backbone 3′ or 5′ exon replacement cassettes to exclude long-distance effects of folding of the vector backbone, again using the webservers Mfold and RNAfold and subjected to further design as described below. One or 2 mismatch nucleotides were introduced into binding domain sequences at every 20–25 nucleotides to prevent effects triggered by long double-stranded RNA (reviewed by Chalupnikova et al.^84^). When structured helical domains were present in the RNA secondary structures of binding domains, C-to-U or A-to-G base exchanges that trigger GU or UG wobble base pairs with the target were introduced to resolve duplexes and promote an unstructured conformation.

### Sequence Conservation Analysis

Sequence conservation of HIV binding domain target regions were determined with the AnalyzeAlign software^33^ using the filtered web LANL database alignment in HIV-1, for either all subtypes or major subtypes. The nucleotide range number refers to residues of HXB2 (GenBank: K03455). A 95% cutoff for calculating the frequency by position was used. For finding variants, the consensus of the alignment was used as master sequence. Nucleotide alignments of pNL4.3 target sequences with subtype B consensus sequence were performed with BLAST Needleman-Wunsch global alignments.^34^ Gaps within consensus sequences were replaced with N for any nucleotide prior to the alignments.

### Cells and Chemicals

293T cells were maintained at 37°C and 5% CO_2_ in DMEM (GIBCO/Life Technologies) supplemented with 10% fetal bovine serum (GIBCO, Life Technologies) and penicillin 100 U/mL and streptomycin 100 μg/mL final concentration (GIBCO, Life Technologies).

Thiazolyl Blue Tetrazolium Salt (3-[4,5-Dimethyl-2-thiozolyl-2,5-diphenyl-2H-tetrazolium bromide] [MTT]) (M5655, Sigma) was resuspended in PBS, filtered through a 0.22 μm filter, and stored in aliquots of 5 mg/mL at −20°C. GCV (G2536, Sigma) was resuspended in 0.1 M HCl to a concentration of 39.6 mM, and aliquots were stored at −20°C.

### Plasmids, Cloning, and Transfections

3′ and 5′ exon replacement cassettes were subcloned into the pVAX-1 backbone (Invitrogen) using *Spe*I and *Bbs*I. HIV binding domains were synthesized by gene synthesis (Life Technologies, Invitrogen) and subcloned into 3′ exon replacement cassettes in the pVAX-1 backbone (Invitrogen) using *Nhe*I and *Mlu*I (Thermo Scientific) restriction enzyme digestion and subcloning. The *Mlu*I site in the pVAX-1 backbone at position 30 was removed by ethidium bromide-mediated partial digestion prior to inserting the HIV binding domains into the 3′exon replacement cassettes. HIV binding domains were inserted into the 5′ exon replacement cassettes in the pVAX-1 backbone using KpnI and *Bbv*CI (New England Biolabs) restriction enzyme digestion and subcloning.

The infectious HIV clone pNL4-3 was obtained from Dr. Malcom Martin through the AIDS Research and Reference Reagent Program, Division of AIDS, National Institute of Allergy and Infectious Disease (NIAID), National Institutes of Health (NIH).^85^

Plasmid DNA was transfected into 293T cells with the Trans-IT-LT1 Transfection reagent (Mirus) in DMEM (GIBCO/Life Technologies) free from fetal bovine serum and antibiotics.

### PCR and Sequencing

Ten nanograms of template cDNA was amplified by two-step PCR with 1.25 U of GoTaq DNA polymerase (Promega) in a PCR containing 5× GoTaq Reaction Buffer (Promega), 2.5 mM PCR Nucleotide mix (Promega), and 500 nM of forward and reverse primers (see Table S3 for primer sequences). Two-step PCR cycling conditions to detect 3′exon replacement RNA *trans*-splicing amplicons were as follows: 95°C for 2 min, 30 cycles of 95°C, 30 sec, 55°C, 30 sec, 72°C, 30 sec (step 1), followed by another 30 cycles of the step 1 cycling conditions above (step 2). A final extension step was performed at 72°C for 5 min, followed by hold at 4°C. PCR products were visualized on 2% agarose gels, and PCR fragments were purified with the QIAquick PCR Purification Kit (Qiagen) according to the manufacturer’s instructions (Qiagen). The presence of RNA *trans*-splicing junctions in purified PCR products were confirmed by Sanger sequencing with the same primer pairs used as in the two-step PCR. RNA *trans*-splicing amplicons after 5′ exon replacement were detected by two-step PCR as above but with 25 cycles of steps 1 and 2.

### qRT-PCR

Total cellular RNA was extracted with the RNeasy Mini Kit (Qiagen) according to the manufacturer’s instructions. Extracted RNA was treated with DNase I (New England Biolabs) for 20 min at 37°C. The DNase was inactivated by the addition of EDTA to a final concentration of 5 mM and incubated for 10 min at 75°C. One microgram of DNase-treated RNA was reverse transcribed using the High Capacity cDNA Reverse Transcription Kit (Applied Biosystems) according to the manufacturer’s instructions. cDNA to an equivalent of 10 ng of DNase-treated RNA was used as a template in qPCR. The qPCRs were as follows: 2× TaqMan Fast Advanced Master Mix (Applied Biosystems), 500 nM forward and reverse primers, and 150 nM probe (see Table S3 for primer and probe sequences). The PCR products were amplified at 50°C for 2 min, 95°C for 20 s, and 50 cycles of 95°C for 3 s and 60°C for 30 s on a 7500 Fast Real Time PCR System (Applied Biosystems). A Ct value of 40 was used as a cutoff for background and mis-priming, and values above Ct 40 were omitted during the analysis.

### Analysis of Cell Viability by the MTT Assay

293T cells (2 × 10^4^) were plated per well in 96-well plates on day 1 and transfected with 100 ng pNL4.3 and RNA *trans*-splicing construct on days 2 and 3, respectively. 293T cells were treated with one or two doses of 100 μM GCV during the 2 following days. Cell viability was assayed on day 8 by the MTT assay on the basis of a previously published method.^86^ MTT was added to each well to a final concentration of 0.5 mg/ml and incubated at 37°C, 5% CO_2_ for 2 hr. Following this, the MTT-containing medium was aspirated, and the formazan crystals were resuspended in acidified (0.04 N HCl) isopropanol/6% Triton X-100 and incubated for 15 min at room temperature to inactivate HIV viral particles. Absorbance was read at 540 and 690 nm on a Multiskan Ascent absorbance plate reader, and the 690 nm reading was subtracted from the 540 nm reading for each well. In Figure S3, absorbance was measured at 595 and 655 nm on a iMark Microplate Reader, BioRad. The 655 nm reading was subtracted from the 595 nm reading for each well.

### Statistical Analysis

Statistical analysis was performed with Microsoft Excel 2010 software using the “t test: two sample assuming unequal variances” function, with p values shown for two-tailed analysis.

## Author Contributions

Conceptualization, C.K.I., S.P., V.P., A.M.L.; Methodology, C.K.I., S.P., V.P., A.M.L.; Investigation, C.K.I., S.P, S.M., V.P.; Resources, V.P.; Writing-Original Draft, C.K.I., A.M.L.; Writing-Review & Editing, C.K.I., S.P., S.M., V.P., A.M.L.; Funding Acquisition, V.P., A.M.L.; Supervision, V.P., A.M.L.

## Conflicts of Interest

S.P. and V.P. declare competing financial interests. A patent application covering the design of *trans*-splicing RNA is pending.

## Supplemental Information

Document S1. Figures S1–S3 and Tables S1–S3

Document S2. Article plus Supplemental Information
