# Supplementary material for: Expression of Herpes Simplex Virus Thymidine Kinase/Ganciclovir by RNA Trans-Splicing Induces Selective Killing of HIV-Producing Cells
Source: Mol Ther Nucleic Acids. 2017 Mar 14;7:140–54. doi: 10.1016/j.omtn.2017.03.004 (PMC5415956; doi:10.1016/j.omtn.2017.03.004)
Supplement: Document S1. Figures S1–S3 and Tables S1–S3 [file mmc1.pdf]

OMTN, Volume 7

Supplemental Information

Expression of Herpes Simplex Virus Thymidine Kinase/Ganciclovir by RNA *Trans*-Splicing Induces Selective Killing of HIV-Producing Cells

Carin K. Ingemarsdotter, Sushmita Poddar, Sarah Mercier, Volker Patzel, and Andrew M.L. Lever

Supplementary figure 1.

a

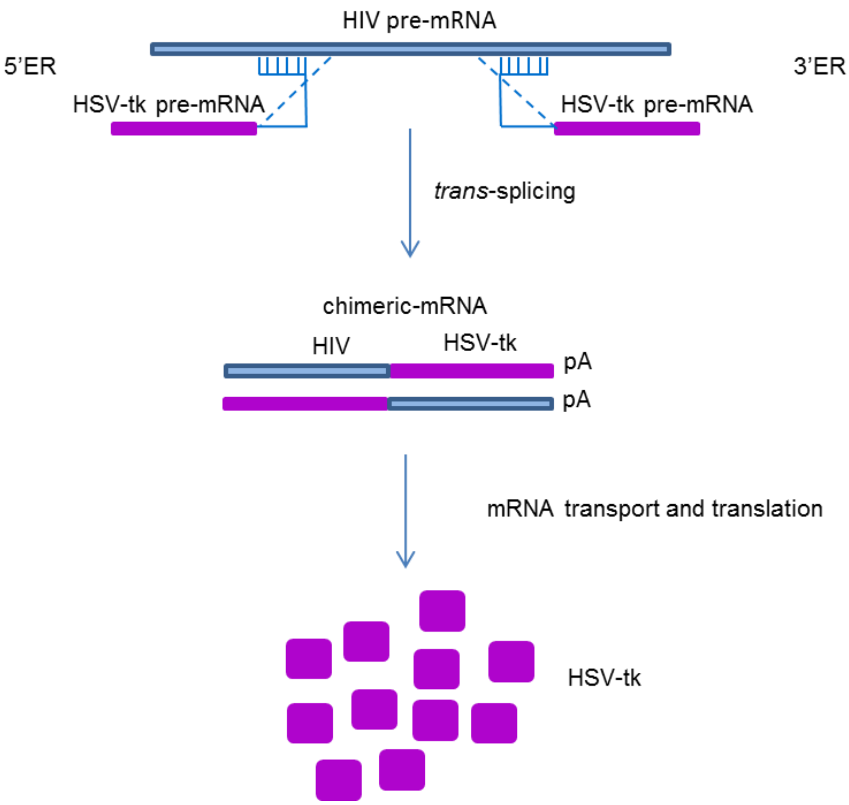

b

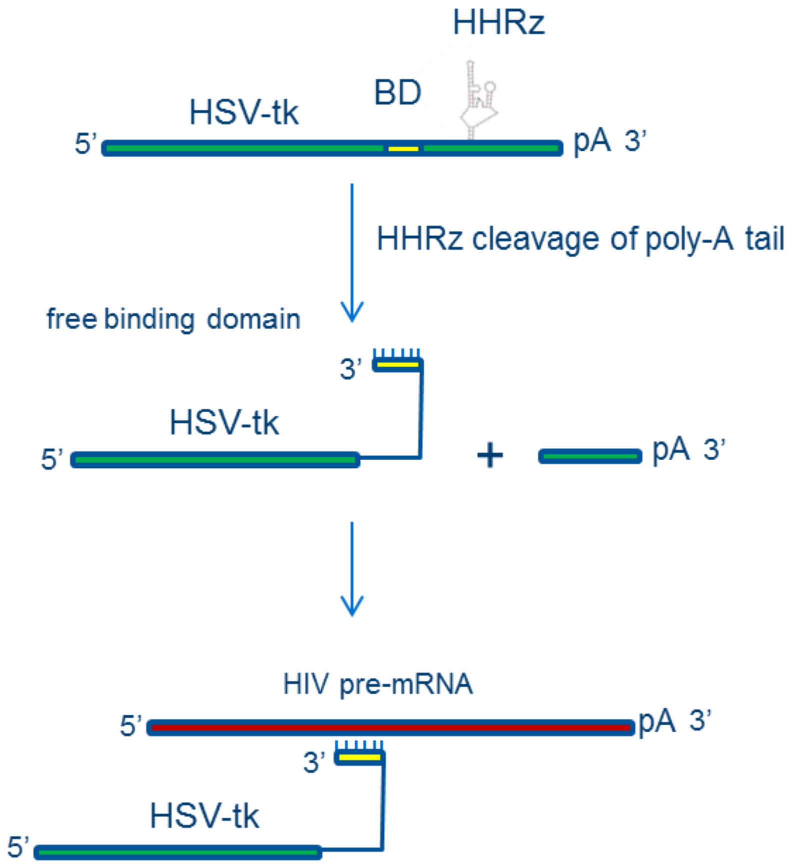

Supplementary figure 1. Schematic diagram of 3'exon replacement and 5'exon replacement.

(a) RNA *trans*-splicing between HSV-tk pre-mRNA (purple) by 5' exon replacement (5'ER) or 3' exon replacement (3'ER) to HIV pre-mRNA (blue). RNA *trans*-splicing lead to the generation of a chimeric RNA transcript that will be transported to the cytoplasm where it will be translated and processed into HSV-tk protein.

(b) Schematic diagram of HHRz self-cleavage. The HHRz[1] sequence is located downstream of HSV-tk and the binding domain (yellow). HHRz RNA self-cleavage will lead to the removal of the polyA tail and exposure of the binding domain. The HSV-tk *trans*-splicing molecule (green) will anneal to the HIV pre-mRNA (red) via the binding domain prior to the RNA *trans*-splicing reaction as shown in Supplementary figure 1a.

1. Saksmerprome, V, Roychowdhury-Saha, M, Jayasena, S, Khvorova, A, Burke, DH (2004). Artificial tertiary motifs stabilize trans-cleaving hammerhead ribozymes under conditions of submillimolar divalent ions and high temperatures. *RNA* **10:** 1916-1924.

Supplementary figure 2.

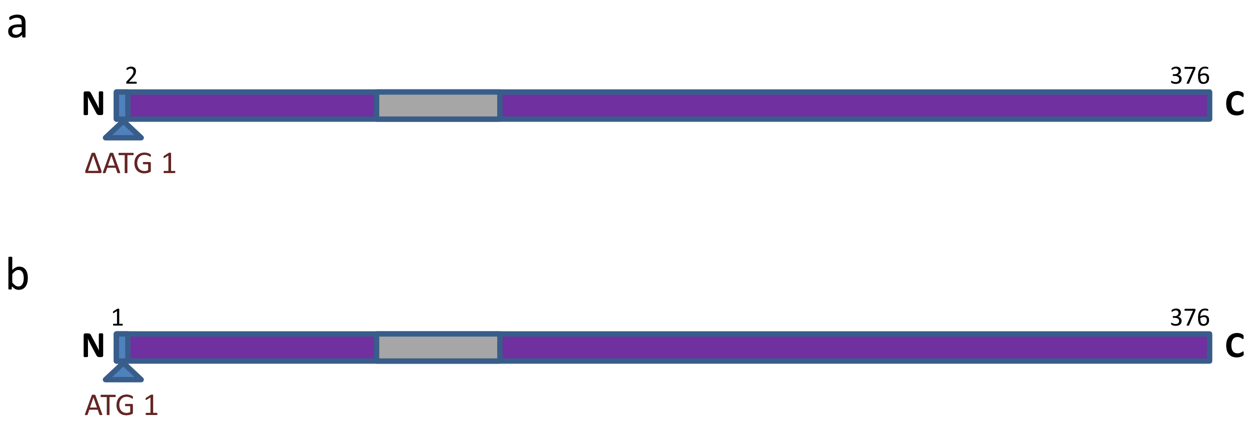

Supplementary figure 2. Diagrams of HSV-tk. (a) Diagram of HSV-tk for 3'exon replacement. The first ATG of HSV-tk is deleted and HSV-tk starts at amino acid 2. An introduced mini-intron is shown in grey. (b) Diagram of HSV-tk for 5'exon replacement. The first ATG is present at amino acid position 1. The mini intron is outlined in grey as above. The numbering refers to amino acid numbering in HSV-tk.

Supplementary figure 3.

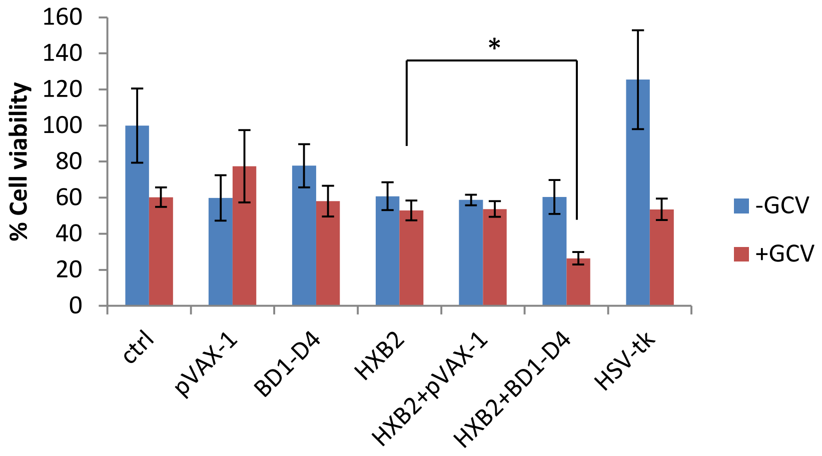

Supplementary figure 3. Cell viability after RNA *trans*-splicing using HXB2 as HIV-1 target strain.

Cell viability was assessed by MTT assay. 293T cells were sequentially transfected with HXB2 (SVC21) and RNA *trans*-splicing construct (BD1-D4) twenty-four hours apart, treated with 100µM GCV on day 1 and 2 after transfection with BD1-D4 (n=8 wells per experimental condition). Cell viability was assessed on day 8, five days post-transfection with the RNA *trans*-splicing construct. Error bars represent the standard deviation (STDEV) of the average % cell viability. P values (two-tailed) are shown for t-test: two sample assuming unequal variances; *$p \leq 0.05$)

Supplementary Table 1.

a

Binding domain	Splice site target	HIV transcript target	HIV-1 pNL4.3 BD target seq.	HIV splice site location (end of exon)	Nts between binding domain and splice site
BD-D1a	D1a	Cryptic splice site	4781-4830	4721	59
BD1-D4	D4	Vpr, Tat, Rev, Nef, Novel	6141-6204	6044	96
BD2-D4	D4	Vpr, Tat, Rev, Nef, Novel	6194-6240	6044	149
BD-LD1/2,env<br>BD-LD1/2,nef	LD1/LD2	Computer algorithm predicted	9302-9339	LD1-9242,<br>LD2-9287	59 (LD1)<br>14 (LD2)

b

Binding domain	Splice site target	HIV transcript target	HIV-1 pNL4.3 BD target seq.	HIV splice site location (end of intron)	Nts between binding domain and splice site
BD-EA1/2	EA1/EA2	Computer algorithm predicted	1773-1820	EA1-2139,<br>EA2-2258	EA1-318,<br>EA2-437
BD-A3	A3	Tat	4780-4829	5776	946
BD-A5	A5	Env/Vpu<br>Rev<br>Nef	5899-5947	5975	27
BD-A7	A7	Vpr, Tat, Rev, Nef, Novel	8188-8235	8368	132
BD-A8	A8	Novel Cryptic	8896-8943	9161	217

Supplementary Table 1. Table of RNA *trans*-splicing constructs with target information for 3’exon replacement in a, and 5’exon replacement in b.

Supplementary Table 2.

Number of HIV-1 sequences (filtered web)	Binding domain	HIV-1 target coordinates in HXB2	Number of mutations in max % of sequences	Max % of sequences
3944	BD1-D4	Vpu 84-147	30	12.75
3944	BD2-D4	Vpu 137-183	32	21.6
4632	BD-A7	Env 1974-2021	7	14.79
2445	BD-A3	Pol 551-600	6	27.55
2712	BD-A5	Tat 70-118	8	19.72
4553	BD-A8	Nef 110-157	33	9.92
HIV-1 sequences (major subtypes A, B, C, D, F, G, CRF01, CRF02)	Binding domain	HIV-1 target coordinates in HXB2	Number of mutations in max % of sequences	Max % of sequences
2960	BD1-D4	Vpu 84-147	23	10.13
2960	BD2-D4	Vpu 137-183	30	22.62
3794	BD-A7	Env 1974-2021	5	15.43
1841	BD-A3	Pol 551-600	6	30.17
1885	BD-A5	Tat 70-118	8	18.77
	BD-A8	Nef 110-157	33	12.52
HIV-1 sequences (Subtype B)	Binding domain	HIV-1 target coordinates in HXB2	Number of mutations in max % of sequences	Max % of sequences
1341	BD1-D4	Vpu 84-147	7	20.44
1341	BD2-D4	Vpu 137-183	27	20.16
1563	BD-A7	Env 1974-2021	3	19.25
773	BD-A3	Pol 551-600	4	34.44
802	BD-A5	Tat 70-118	4	21
1627	BD-A8	Nef 110-157	9	14.55

Supplementary Table 2. Summary of sequence variant analysis of HIV-1 binding domain target sequences. Sequence variants of HIV-1 binding domain target sequences were analysed using AnalyzeAlign as described in Material and Methods.

Supplementary Table 3.

Name of primer	F or R	Primer sequence	Usage
3'ER reverse	R	GGTTCTCCTCCACGTCTC	to detect t.s junction by conventional PCR/sequencing
5'ER forward	F	TACCGACGATCTGCGAC	to detect t.s junction by conventional PCR/sequencing
5’ER BD-A3 reverse	R	TCGAGTAACGCCTATTCTG	to detect t.s junction by conventional PCR
5'ER BD-A7 reverse	R	ACCAATATTTGAGGGCTTC	to detect t.s junction by conventional PCR/sequencing
5'ER BD-A8 reverse	R	TTATCTGGCTCAACTGGTACTA	to detect t.s junction by conventional PCR/sequencing
5’ER BD-A5 reverse	R	TGCTTTGATAGAGAAGCTTGATGAG	to detect t.s junction by conventional PCR/sequencing
3'ER BD1/2-D4 forward	F	GAGACAGCGACGAAGAGCTCAT	to detect RNA trans-splicing transcripts by qRT-PCR, conventional PCR, sequencing
3'ER reverse	R	CAGGGTTCTCCTCCACGTCTC	to detect RNA trans-splicing transcripts by qRT-PCR
5'ER BD-A7 reverse	R	CACCTTCTTCTTCTATTCCTTCGG	to detect RNA trans-splicing transcripts by qRT-PCR
5'ER BD-A3 reverse	R	CCTCTGTCGAGTAACGCCTATTCT	to detect RNA trans-splicing transcripts by qRT-PCR
5'ER BD-A5 reverse	R	AGTCTGACTGTTCTGATGAGCTCTTC	to detect RNA trans-splicing transcripts by qRT-PCR
5'ER BD-A8 reverse	R	TCTACCTTATCTGGCTCAACTGGTACTA	to detect RNA trans-splicing transcripts by qRT-PCR
5'ER forward	F	TGGCGCGCACGTTTG	to detect RNA trans-splicing transcripts by qRT-PCR
3'ER probe	Probe	6FAM-GAAGCGGAGCTACTAACTTCAGCCTGCTGA-BHQ1	to detect RNA trans-splicing transcripts by qRT-PCR
5'ER probe	Probe	6FAM-CGGGAGATGGGGGAGGCGAA-BHQ1	to detect RNA trans-splicing transcripts by qRT-PCR

Supplementary Table 3. Primers used for PCR, qRT-PCR and sequencing.
